# Giant cyamella: a rare sesamoid bone

**DOI:** 10.1590/0100-3984.2015.0240

**Published:** 2017

**Authors:** Márcio Luís Duarte, André de Queiroz Pereira Silva, Simone Botelho Alvarenga, José Luiz Masson de Almeida Prado, Luiz Carlos Donoso Scoppetta

**Affiliations:** 1 WebImagem, São Paulo, SP, Brazil.; 2 CADI Diagnóstico, Imperatriz, MA, Brazil.; 3 Axial Medicina Diagnóstica, Belo Horizonte, MG, Brazil.; 4 Hospital São Camilo, São Paulo, SP, Brazil.

Dear Editor,

Here, we report the case of a 46-year-old male who presented with a three-year history of pain in the right knee. He stated that he had experienced no trauma or torsion, had not undergone any surgery, and did not engage in sports. In the physical examination, he tested positive for a meniscal tear. Magnetic resonance imaging (MRI) revealed a giant cyamella; complex rupture of the body and anterior horn of the lateral meniscus; and chondral lesions in the lateral femorotibial compartment (Figure 1).

**Figure 1:**
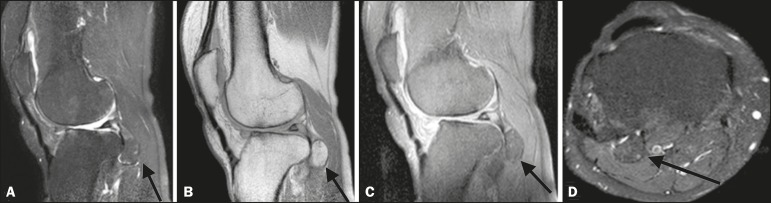
Non-contrast-enhanced MRI scans. T2-weighted spectral presaturation inversion recovery (SPIR) sequence (**A**) and proton-density (PD) sequence (**B**), both acquired in the sagittal plane, showing a voluminous ossified mass, measuring 2.2 × 1.7 × 1.5 cm (arrows), in the popliteal tendon. Sagittal PD-SPIR sequence (**C**) and axial T2-weighted SPIR sequence (**D**) showing a voluminous ossified mass in the popliteal tendon (arrows).

Sesamoid bones are accessory ossicles located in the tendons and muscles; their function is to facilitate the physiological movement of the tendon, although they can, in some cases, cause disease^(1)^. Most sesamoid bones are located in the lower limbs^(2)^. Embryologically, sesamoid bones are generally more common in the fetus; with skeletal growth and bone maturation, many sesamoid bones fuse^(2,3)^. In humans, the largest sesamoid bone is the patella^(2)^.

The popliteal tendon typically originates at the lateral femoral condyle, its muscle inserting into the posterior surface of the tibia above the soleal line^(4)^. The sesamoid bone that can exist in the tendon of the popliteal muscle is known as the cyamella, popliteal fabella, fabella distalis, or sesamoideum genu inferius laterale^(5)^. It is often confused with the fabella, which is within the lateral head of the gastrocnemius muscle^(5)^.

Although the cyamella is common in other primates, it is quite rare in humans, and, when it occurs, it can articulate with the lateral condyle of the tibia and be quite near the head of the fibula^(3,4)^. However, it does not have a well-defined function^(6)^. It resides as an accessory ossicle in the popliteal tendon itself or at the intersection between the tendon and the muscle^(6,7)^; its size can vary considerably^(3)^, and it should be clearly distinguished from free bodies, calcifications, osteophytes, and the fabella, as well as from osteochondromatosis^(3)^ and avulsion of the popliteal tendon^(7)^.

A diagnosis of cyamella can be established through various imaging modalities, such as X-ray, computed tomography, and MRI^(3)^. On T1-, T2-, and T2*-weighted MRI scans, a cyamella appears as an ossicle with low signal intensity along its borders^(6)^. A computed tomography scan can reveal fat within the ossicle^(6)^. Due to the rarity of cyamella, its characterization and the exclusion of other potential diagnoses are of particular clinical relevance^(3)^.

In patients with lateral knee pain, physicians should bear in mind the possibility of cyamella as the cause of the pain^(1)^. Cyamella typically does not have pathological implications, although cyamella-associated pain has been described^(1)^. Because of the rarity of the diagnosis, there is no consensus regarding the treatment of cyamella, which should therefore be treated on a case-by-case basis, the symptoms and imaging findings being taken into account^(3)^.

## References

[1] Rehmatullah N, McNair R, Sanchez-Ballester J (2014). A cyamella causing popliteal tendonitis. Ann R Coll Surg Engl.

[2] Akansel G, Inan N, Sarisoy HT (2006). Popliteus muscle sesamoid bone (cyamella): appearance on
radiographs, CT and MRI. Surg Radiol Anat.

[3] Khanna V, Maldjian C (2015). The cyamella, a lost sesamoid: normal variant or posterolateral
corner anomaly?. Radiol Case Rep.

[4] Reddy S, Vollala VR, Rao R (2007). Cyamella in man - its morphology and review of
literature. Int J Morphol.

[5] Keats TE, Anderson MW (2007). Atlas of normal roentgen variants that may simulate disease.

[6] Munk PL, Althathlol A, Rashid F (2009). MR features of a giant cyamella in a patient with osteoarthritis:
presentation, diagnosis and discussion. Skeletal Radiol..

[7] Jadhav SP, More SR, Riascos RF (2014). Comprehensive review of the anatomy, function, and imaging of the
popliteus and associated pathologic conditions. Radiographics.

